# Revolutionizing endocarditis diagnosis: AI meets metagenomics for rare *Bartonella* detection

**DOI:** 10.1097/MS9.0000000000003942

**Published:** 2025-09-23

**Authors:** Awaisi Muhammad Abdur Rehman, Muhammad Hashim Rizwan, Maliha Khalid, Muhammad Talha, Aminath Waafira

**Affiliations:** a Department of Medicine, King Edward Medical University, Lahore, Pakistan; b Department of Medicine, Jinnah Sindh Medical University, Karachi, Pakistan; c King Edward Medical University, Lahore, Pakistan; d School of Medicine, The Maldives National University, Malé, Maldives

**Keywords:** artificial intelligence, *Bartonella* endocarditis, infectious disease diagnosis, metagenomic sequencing

## Abstract

*Bartonella* endocarditis is notoriously challenging to diagnose due to its insidious onset, nonspecific symptoms, and the limitations of conventional culture-based techniques. Artificial intelligence (AI)-driven metagenomic next-generation sequencing (mNGS) represents a transformative diagnostic approach by integrating machine learning algorithms with culture-independent sequencing data to improve accuracy and sensitivity. This technique overcomes barriers such as culture bottlenecks and post-surgical sample limitations, achieving high diagnostic specificity while enabling the detection of rare or novel pathogens. Despite challenges including limited reference databases, contamination risks, and cost-related barriers in low-resource settings, AI-enhanced metagenomics offers a promising path toward faster and more precise diagnosis of *Bartonella* endocarditis. Its integration into clinical workflows, supported by continuous algorithm development, cost optimization, and standardized protocols, has the potential to improve patient outcomes in rare infectious diseases.

*To the Editor,*

*Bartonella* species are gram-negative bacteria that cause a wide range of clinical manifestations, like localized lymphadenopathy and severe systemic diseases such as cat-scratch disease, Carrion’s disease, trench fever, and endocarditis. These pleomorphic coccobacillary organisms are transmitted by arthropod vectors and infect erythrocytes, endothelial cells, and various macrophage-type cells, including microglial cells and dendritic cells^[1]^. *Bartonella* endocarditis has an insidious onset and non-specific symptoms. It presents a particularly challenging diagnosis due to the difficulty in culturing the causative agents from blood^[2]^. Rapid and accurate diagnostic tools are critically needed due to possible cardiac damage and fatal outcomes without treatment, and artificial intelligence (AI)-driven metagenomics fulfills this need.

Metagenomics is a computational method that enables researchers to reconstruct microbial genomes directly from a given sample. Studies indicate that metagenomics and next-generation sequencing (NGS) have been used for the identification of central nervous system infections (meningitis, encephalitis) caused by pathogens like *Cryptococcus, Aspergillus*, and *Mycobacterium tuberculosis*^[3]^. AI-driven metagenomics is revolutionary because it integrates machine learning algorithms with data obtained from NGS. AI algorithms are important because they process the detailed datasets generated by metagenomic sequencing. This makes data processing more efficient and also ensures that different microorganisms are accurately identified. Computational tools are great at identifying subtle patterns and predicting microbial functions. These tools are even capable of uncovering novel microbial species and genes that conventional bioinformatics pipelines would find intractable^[4]^.

Infective endocarditis is difficult to diagnose due to the inconspicuous nature of *Bartonella* species, like in blood-culture-negative endocarditis. These intracellular organisms are difficult to isolate and diagnose using conventional techniques such as valve culture and polymerase chain reaction (PCR). AI-driven metagenomics next-generation sequencing (mNGS) has revolutionized the diagnosis of infective endocarditis as it has achieved a sensitivity of 82.4% and a specificity of 100%, which are markedly high in comparison to valve culture and microscopy, but has a little lower sensitivity than in blood culture^[5]^. This technique mainly uses culture-independent sequencing of total genetic material, which overcomes persistent limitations such as the culture bottleneck and specimen-specific variability^[6]^. Usually, conventional tests fail to detect any remains of causative agents after valve replacement surgery; in such cases, metagenomics (mainly its branch metataxonomics) is helpful in detecting the infective agent.

AI-driven mNGS has transformative potential for the diagnosis of *Bartonella* infections, yet it has limitations. A primary challenge is the lack of availability of reference databases, because it is hard to reference sequence a vast amount of data, which limits the accurate detection of pathogens^[7]^. Additionally, the difficulty in differentiating true pathogens from background contaminants contributes to false positives, while low-abundance targets may be missed which resulting in false negatives. In resource-limited settings, scalability and accessibility are restricted due to high financial and computational costs. Most clinical laboratories lack the bioinformatics expertise required to analyze complex mNGS data.

In conclusion, the successful integration of mNGS into standard diagnostic workflows is quite necessary. This requires the continuous development and rigorous clinical validation of emerging AI models, designed to accurately interpret complex data sequencing and detect true pathogens. Furthermore, different strategic measures are required to make it cost-effective and establish standardized interpretation protocols. By adopting these developments, rare *Bartonella* endocarditis can be diagnosed much more quickly and accurately, improving patient outcomes and treatment in the long run. This letter to editor is in compliance with the TITAN guideline^[8]^.

## Data Availability

No data were generated for this manuscript.

## Research registration unique identifying number (UIN)

Name of the registry: NA Unique Identifying number or registration ID: NA Hyperlink to your specific registration (must be publicly accessible and will be checked): NA
